# Drug-Loaded Hydrogels for Intraocular Lenses with Prophylactic Action against Pseudophakic Cystoid Macular Edema

**DOI:** 10.3390/pharmaceutics13070976

**Published:** 2021-06-28

**Authors:** Nadia Toffoletto, Madalena Salema-Oom, Soledad Anguiano Igea, Carmen Alvarez-Lorenzo, Benilde Saramago, Ana Paula Serro

**Affiliations:** 1 Centro de Química Estrutural, Instituto Superior Técnico, University of Lisbon, Avenue Rovisco Pais, 1049-001 Lisbon, Portugal; b.saramago@tecnico.ulisboa.pt (B.S.); anapaula.serro@tecnico.ulisboa.pt (A.P.S.); 2 Centro de Investigação Interdisciplinar Egas Moniz, Instituto Universitário Egas Moniz, Quinta da Granja, Monte de Caparica, 2829-511 Caparica, Portugal; moom@egasmoniz.edu.pt; 3 HGBeyond Materials Science S.L., Edificio EMPRENDIA, 15782 Santiago de Compostela, Spain; research@hgbeyond.com; 4 Departamento de Farmacología, Farmacia y Tecnología Farmacéutica, I + D Farma (GI-1645), Facultad de Farmacia and Health Research Institute of Santiago de Compostela (IDIS), Universidade de Santiago de Compostela, 15782 Santiago de Compostela, Spain; carmen.alvarez.lorenzo@usc.es

**Keywords:** therapeutic ophthalmic lenses, posterior segment diseases, drug release, anti-inflammatory drug, molecular imprinting, functionalized hydrogels

## Abstract

Pseudophakic cystoid macular edema (PCME), caused by chronic inflammation, is the most common cause of visual impairment in the medium-term after cataract surgery. Therefore, the prophylactic topical administration of combined steroidal and non-steroidal anti-inflammatory drugs is commonly done. Drug-eluting intraocular lenses (IOLs) gained interest as an efficient way to overcome the compliance issues related to the use of ocular drops without the need for additional surgical steps. The incorporation of functional monomers and molecular imprinting were herein applied to design hydrogels suitable as IOLs and able to co-deliver steroidal (dexamethasone sodium phosphate) and non-steroidal (bromfenac sodium) drugs. The incorporation of N-(2-aminopropyl) methacrylamide (APMA) increased the drug uptake and improved the in vitro release kinetics. Imprinting with bromfenac resulted in a decreased drug release due to permanent drug bonding, while imprinting with dexamethasone increased the amount of dexamethasone released after dual-drug loading. The application of a mathematical model to predict the in vivo drug release behavior suggests the feasibility of achieving therapeutic drug concentrations of bromfenac and dexamethasone in the aqueous humor for about 2 and 8 weeks, respectively, which is compatible with the current topical prophylaxis after cataract surgery.

## 1. Introduction

Cataracts are the leading cause of vision loss in the elderly population worldwide [1,2]. The pathology consists in the opacification of the natural crystalline lens and is commonly treated by surgical replacement of the damaged crystalline with an artificial intraocular lens (IOL). Although cataract surgery is currently one of the most cost-effective procedures in healthcare [3], some post-surgical complications may occur. The most common cause of visual impairment in the medium-term after IOL implant is pseudophakic cystoid macular edema (PCME), also called Irvine-Gass syndrome [4,5]. PCME is caused by the presence of chronic inflammation, and it affects up to 3.6% of cataract patients in its symptomatic acute form [6]. Its incidence increases with the concomitance of pre-existing risk factors, such as contralateral PCME, diabetes mellitus, uveitis, history of retinal vein occlusion and retinal degeneration, macular degeneration, retinopathy, epiretinal membranes, and the use of prostaglandins [6]. In most cases, PCME can be solved with adequate pharmacological treatment. However, in non-responding patients, it may become a serious issue and lead to permanent vision loss [4,5,7]. For this reason, the prophylactic topical administration of anti-inflammatory drugs is commonly done after surgery [8]. As most cataract patients are elderly people, prescription of ocular drops may raise compliance issues. Moreover, due to the low penetration of drugs through the corneal epithelium and the important drug loss by lacrimation, other methods are needed for the intraocular delivery of less permeative drugs [9]. In this context, the development of drug-loaded IOLs would constitute an efficient way to administer drugs without the need for additional steps during surgery or during patient recovery. These devices are not yet present on the market, although their development has been pursued by several researchers [10]: therapeutic IOLs have been designed for the sustained release of antibiotics [11,12,13,14,15], anti-inflammatory drugs [13,14,16,17], immunosuppressants [18] and anti-proliferative drugs for the prevention of the posterior capsule opacification (PCO) [19,20,21,22,23].

As PCME presents a peak of incidence at 4–6 weeks after surgery, the administration of anti-inflammatory drugs is usually prescribed for one month [6,8]. Topical steroids were considered the classic prophylaxis for PCME in cataract patients [24]. However, higher efficacy has been reported for the combined use with topical non-steroidal anti-inflammatory drugs (NSAIDs) [4]. Indeed, co-administration of NSAIDs (e.g., nepafenac or bromfenac) and corticosteroids (e.g., dexamethasone sodium phosphate) has been shown to lower the incidence of PCME [24]. In the ESCRS PREMED study, an international randomized controlled clinical trial [11], the use of topical bromfenac, dexamethasone sodium phosphate or a combined therapy was investigated, and again the incidence of PCME was lower in the case of the combined treatment.

Several strategies have been suggested in the last decade to tune drug release from ocular devices. Molecular imprinting is an innovative technique that has been demonstrated to prolong drug release from several therapeutic ophthalmic lenses [25,26,27,28]. It consists of the non-covalent association between a drug, acting as a template during the polymerization step and functional monomers incorporated into the polymeric matrix. After polymerization and drug removal, tailored active sites remain imprinted in the polymer, generating an oriented structure with a high affinity for the template drug. The drug can be re-loaded into the polymer by soaking, and, due to the interaction with the oriented functional monomers, a higher drug loading and a slower release profile can be achieved. The type and concentration of the functional monomers tune the drug affinity to the polymer and the release kinetics. Therefore, optimization is necessary for each combination of drug and monomer [29,30].

The aim of the present work is to design functionalized and/or imprinted drug-loaded hydrogels for the prevention of PCME. To our knowledge, the development of dual-loaded IOLs, able to simultaneously deliver steroidal and non-steroidal anti-inflammatory drugs, is still an unmet medical need. Two hydrophilic drugs were selected for their proven efficacy in the prevention of PCME: bromfenac sodium, an NSAID, and dexamethasone sodium phosphate, a corticosteroid [31,32].

**Figure 1:**
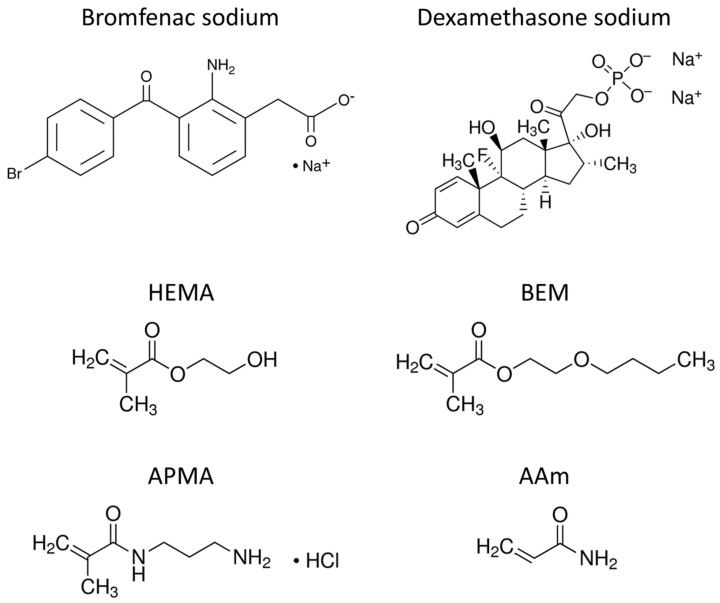
Molecular structure of the two selected drugs (bromfenac sodium and dexamethasone sodium), the hydrogel backbone monomers (HEMA and BEM) and the functional monomers (APMA and AAm).

Two hydrophilic monomers commonly found in commercial IOLs were selected to produce the hydrogel: HEMA (2-hydroxyethyl methacrylate), a component of acrylic IOLs, and BEM (2-butoxyethyl methacrylate), which is known to increase the free-volume of the copolymer and the IOL flexibility, thus requires a smaller incision during implant surgery [33]. Functionalization was done with two monomers: APMA (N-(2-aminopropyl) methacrylamide) and AAm (acrylamide), considered as potentially suitable due to the interaction between the positive charge that they acquire in aqueous solution and the negative charge of the selected drugs in solution, at pH 7.4. The molecular structures of the drugs and the monomers are presented in Figure 1.

## 2. Materials and Methods

### 2.1. Materials

Dexamethasone sodium phosphate (CAS 2392-39-4) and bromfenac sodium (CAS 91714-93-1) were purchased from Carbosynth (Compton, UK). EGDMA (CAS 97-90-5), AIBN (CAS 78-67-1), HEMA (CAS 868-77-9) and AAm (CAS 79-06-1) were purchased from Sigma-Aldrich (Steinheim, Germany). BEM (CAS 13532-94-0) was purchased from abcr (Karlsruhe, Germany) and APMA from PolySciences (Eppelheim, Germany). Distilled and deionized water (18 MΩcm, pH7.7) was obtained from a Millipore system. Methanol HPLC grade (CAS 67-56-1) was purchased from ChemLab (Zedelgem, Belgium) and acetonitrile HPLC grade (CAS 75-05-8) from Carlo Erba Reagents (Val-de-Reuil, France). Phosphate buffer saline (PBS), pH 7.4, was purchased from Sigma–Aldrich (Darmstadt, Germany). Phosphate buffer (pH 6) was prepared with the following composition: NaOH 1.15 mM (VWR—Leuven, Belgium) and KH_2_PO_4_ 10 mM (ITW Reagents—Barcelona, Spain). Porcine eyes, provided by a local slaughterhouse (Compostelana de Carnes S.L.—Santiago de Compostela, Spain), were immersed in PBS solution, transported in an ice bath and used within three hours after collection. Human lens epithelial cells (ATCC-CRL-11421 1 B-3) were purchased from LCG Standards (Barcelona, Spain). Dulbecco’s Modified Eagle’s Medium (DMEM), fetal bovine serum, penicillin, streptomycin, yellow tetrazolium (3-(4,5-dimethylthiazolyl-2)-2,5-diphenyltetrazolium bromide) (MTT), dimethyl sulfoxide (DMSO, ≥99%), isopropanol and hydrochloric acid were purchased from Sigma-Aldrich (Steinheim, Germany). IGEPAL^®^ was purchased from Merck (Darmstadt, Germany).

### 2.2. Molecular Interaction Analysis

The modeling of the drug-monomer interaction was performed to select components potentially suitable for the drug-releasing hydrogels, using the AutoDock Tools 1.5.6 software (MGL Tools, Scripps Research, La Jolla, CA, USA). The 3D model of the molecules was obtained from the PubChem database [34] and files were converted with OpenBabel GUI software (OpenEye Scientific, Santa Fe, NM, USA). Then, each monomer-drug couple was analyzed. Briefly, the drug molecule was selected as ‘macromolecule’, and the monomer as ‘ligand’. The grid was generated with default settings. Molecular docking was performed through the Lamarckian genetic algorithm. The obtained conformations were ranked by energy, and the lowest energy conformation was chosen as a result. All torsions were allowed to rotate during docking. The free energy of interaction, E, and the dissociation constant, Ki, were obtained as output [35].

### 2.3. Hydrogels Synthesis

Nine different hydrogels were prepared, as summarized in Table 1. Group A corresponds to non-imprinted hydrogels, group B to bromfenac-imprinted hydrogels and group C to dexamethasone-imprinted hydrogels. For each group, three different monomer compositions were tested to investigate the influence of APMA and AAm as functional monomers.

The monomer mixtures (3 mL) were stirred for 24 h at room temperature. Then, the solutions were injected into molds made of silanized glass with a 0.5 mm silicone rubber separator. Thermal polymerization was performed through a first step incubation at 50 °C for 12 h, followed by a second step at 70 °C for 24 h. After polymerization, the hydrogels were boiled for 15 min in 900 mL of distilled water. Then, the obtained polymer sheets were cut into 6 mm discs using a biopsy puncher to resemble the size of commercial IOLs and subjected to an additional washing step to remove the unreacted monomers and the drug molecules used as a template in the imprinted networks. The washing procedure was performed by soaking the discs under stirring in distilled water (14 days, renewed twice a day), followed by soaking in ethanol (48 h), NaCl 0.9% (12 h) and distilled water (48 h). During the last step, no peaks associated with the presence of residual monomers or the drug could be detected by UV-vis spectrometric analysis of the washing solution. The obtained discs were then dried at 35 °C for 72 h prior to storage. Dry discs weighed approximately 13 mg.

### 2.4. Drug Loading and Release

Single drug loading was performed to evaluate the interaction between the hydrogels and each drug, the influence of the functional monomers and the effect of molecular imprinting on the release profiles. Non-imprinted hydrogels (A1, A2, A3) and bromfenac-imprinted hydrogels (B1, B2, B3) were loaded by soaking each dry disc (*n* = 3) into 1 mL of bromfenac sodium solution (1 mg/mL in PBS). Non-imprinted hydrogels (A1, A2, A3) and dexamethasone-imprinted hydrogels (C1, C2, C3) were loaded by soaking each dry disc (*n* = 3) into 1 mL of dexamethasone sodium solution (5 mg/mL in PBS). A higher concentration was used for dexamethasone since preliminary studies (data not shown) indicated that the amount of dexamethasone loaded and released from the hydrogels was much lower than that of bromfenac. Soaking was performed at 36 °C for 1 week under agitation at 180 rpm.

After the evaluation of the single-drug release profile, dual drug loading of the best hydrogels was performed by soaking each dry disc (*n* = 3) into 1 mL of dual drug solution (i.e., 1 mg/mL bromfenac sodium + 5 mg/mL dexamethasone sodium in PBS). Soaking was done at different temperatures (4 °C, 36 °C or 60 °C) and soaking times (1, 2 or 4 weeks) to identify the optimal loading conditions.

The release profiles of both single- and dual-loaded hydrogels were evaluated in vitro for 25 days. The drug-loaded hydrogels were removed from the soaking solution, rinsed in distilled water, and gently blotted with absorbent paper. Then, each disc was completely immersed in 3 mL of PBS and placed in a shaker at 36 °C and 180 rpm. At each time point, aliquots of 0.3 mL were collected to be analyzed and were replaced by the same volume of fresh PBS.

### 2.5. Drug Quantification

Drug quantification was performed by UV-Vis spectroscopy (MultiscanGO, ThermoScientific, Porto Salvo, Portugal) in all tests, except when explicitly stated otherwise. The absorbance of bromfenac sodium and dexamethasone sodium in water or PBS was detected respectively at the wavelengths of 268 nm and 242 nm. In turn, in methanol, drugs were detected at 260 nm and 238 nm, respectively. A calibration curve was obtained for each drug, in the various liquid media.

To quantify each drug in dual-drug solutions, the entire spectrum was deconvoluted according to the method previously developed by Kim and Chauhan [36], which considered the dual-drug spectrum as a linear combination of the single-drug spectra. A least-square fit between the dual-drug and single-drug spectra were applied to determine the concentration of each drug.

Due to the difficulties encountered in the application of the deconvolution method to low drug concentrations, chromatographic analysis was used for quantification in the first 48 h of dual drug release in vitro (Section 2.4) and during the ex-vivo testing (Section 2.8). A Waters HPLC apparatus (Milford, MA, USA) with a UV detector was used. A C18 column (4.6 × 150 mm, ACE^®^, Aberdeen, UK) with 5 µm pores was selected. The analysis was performed at room temperature, using as mobile phase a mixture of 0.01 M phosphate buffer (pH 6.0) and acetonitrile (72:28 *v*/*v*), with a flow rate of 1 mL/min. For the in vitro release and ex-vivo testing, the selected injection volumes were 20 µL and 80 µL, respectively. Dexamethasone sodium and bromfenac sodium were quantified at 242 nm and 265 nm, respectively, with a retention time of 4.20 min and 14.65 min. HPLC calibration curves were validated for linearity, accuracy and precision.

All quantification measurements were performed at least in triplicate.

### 2.6. Drug Amount Loaded

To evaluate the total amount of drug loaded in the best materials, hydrogel discs (*n* = 3) were rinsed after loading in a dual-drug solution, gently blotted and placed in glass vials with 3 mL methanol. Each 24 h, the extraction medium was analyzed by UV-vis spectroscopy and replaced by fresh methanol. The amount of drug loaded was quantified using the previously described deconvolution method [36] with calibration curves for each drug in methanol. The analysis was repeated until no drug could be detected in the extraction medium. The total amount of drug extracted corresponded to the sum of the drug amounts quantified at each measurement.

### 2.7. Physical and Mechanical Characterization

To evaluate the liquid uptake of the hydrogels, bare hydrogel discs (*n* = 3) were weighed after the washing and drying procedure. Then, each disc was immersed in 3 mL of PBS at 36 °C under agitation (100 rpm). The weight variation was monitored at *t* = 0.5, 1, 2.5, 5, 8 h and every 24 h until a plateau value was reached. Liquid uptake was calculated by Equation (1), where w_0_ and w_t_ represent the weight of the dry hydrogel and of the hydrogel at time *t*, respectively.

Liquid uptake (%) = [(w_t_ − w_0_)/w_0_] × 100 (1)

The same equation was used to evaluate the swelling of the hydrogels after dual drug loading. In this case, w_t_ of samples (*n* = 3) was measured at *t* = 1 week of soaking in 1 mL of dual drug solution (i.e., 5 mg/mL dexamethasone sodium + 1 mg/mL bromfenac sodium in PBS, 180 rpm, T = 36 °C).

Hydrogels (n ≥ 3) were tested for transmittance after hydration in PBS and after dual drug loading under the previously defined conditions. Transmittance measurements were performed using a UV-vis spectrophotometer (MultiscanGO, ThermoScientific, Porto Salvo, Portugal). The wavelength interval of 200–800 nm was scanned with 1 nm intervals.

The tensile modulus of the hydrogels was evaluated with a TA.XT Express Texture Analyzer (Stable Micro Systems, Godalming, UK). Dog-bone shaped samples (n ≥ 5, thickness ≈0.5 mm, width ≈2.5 mm) were tested after hydration in PBS and after dual drug loading under the previously defined conditions. The test was performed at 0.3 mm/s up to failure, with a trigger force of 0.005 N [28]. Young’s modulus was determined from the initial slope (ε = 0–5%) of the obtained stress-strain curve.

The average mesh size ξ of the hydrogels was calculated by Equation (2), where l_c−c_ is the length of the carbon-carbon bond in the hydrogel backbone, C_F_ is the Flory characteristic ratio, ρ is the dry hydrogel density, M_r_ is the molecular weight of the repeating unit, G′(0) is the zero-frequency shear storage modulus (defined as G′(0) = Young’s modulus × 1/3, assuming a Poisson ratio of 0.5) and φ is the volume fraction of the polymer in the swollen network [37]. The values of those parameters are given in Supplementary Materials Table S1.

(2) $$\xi =l_{c-c}\sqrt{\frac{2{\;C}_{F}\rho \mathrm{RT}}{M_{r}G^{\prime }\left(0\right)}}\varphi^{-1/6}$$

### 2.8. Prediction of In Vivo Efficacy

A previously developed mathematical model was applied to estimate the drug concentration in the aqueous humor over time, after the hydrogel implantation [38]. The input data included the thickness (≈0.45 mm) and diameter (≈6 mm) of the hydrogel discs and the loading conditions, namely, volume (1 mL), the concentration of the loading solution (1 mg/mL bromfenac sodium and 5 mg/mL dexamethasone sodium), and soaking time (t = 1 week)). The partition coefficient K between the hydrogel and the release medium, the effective diffusivity D of the drugs through the hydrogel network, and the drug loss through the cornea were also required for the estimation of the behavior of the hydrogels in vivo.

K was calculated as the ratio between the drug concentration in the hydrogel disc (C_disc_) and in the release medium (C_medium_) at equilibrium (Equation (3)) [39]. With this purpose, dual-loaded A1, A2, B2 and C2 hydrogels were placed in 10 mL PBS and C_medium_ was analyzed weekly until the equilibrium was reached. Then, the drug amount in the hydrogels was extracted with methanol as described above and normalized by the disc volume to obtain C_disc_.

K = C_disc_/C_medium_ (3)

The effective diffusivity D was obtained as previously described by Pimenta et al. [38]. Briefly, considering the hydrogel as a uniform thickness film and applying Fick’s second law (Equation (4)), it is possible to obtain a one-dimensional diffusion equation (Equation (5)) which can be fitted to the in vitro release data. C and A represent, respectively, the drug concentration in the hydrogel and its surface area, while Cr and Vr refer to the concentration and volume of the release medium, respectively.

∂C/∂t = D (∂^2^C/∂y^2^) (4)

−2 D A (∂C/∂y)_y = h_ = Vr (∂Cr/∂t) (5)

The drug permeability through the cornea, P_cornea_, was required to estimate the drug loss through the tissue. The permeability values of bromfenac sodium and dexamethasone sodium were obtained with ex vivo tests which involved Franz diffusion cells and porcine corneas, as described in a previous publication [40]. Briefly, corneas were excised with 2–3 mm of surrounding sclera for support and then placed with the endothelium facing the donor chamber of the Franz cell to simulate the in vivo conditions of the tissue when exposed to intraocular drug sources. A2 and C2 hydrogel discs (*n* = 3), loaded with both bromfenac and dexamethasone, were rinsed from the loading solution, placed in each donor chamber, and covered with 1 mL PBS. The receptor chamber was filled with 6 mL PBS. Franz cells were placed in a bath at 37 °C with magnetic stirring of the receptor chamber. At each time point (0.5, 1, 2, 3, 4, 5, 6 h), 1 mL of PBS was removed from the receptor chamber and substituted with fresh PBS. After the last time point, the volume of the donor chamber was collected for analysis. Drug quantification was performed as explained above (Section 2.5). All samples were filtered (0.22 µm PTFE hydrophilic syringe filters, Scharlau^®^) before testing. The cumulative amounts of permeated drug were fitted to a linear least squares regression (Equation (6)), and the slope of the regression was the steady-state flux J. The P_cornea_ of each drug was obtained as the ratio between J and the drug concentration in the donor chamber at *t* = 6 h (Equation (7)) [41,42].

Cumulative mass permeated/Surface area = J t + q(6)

P_cornea_ = J/[Donor]_t = 6 h_ (7)

The drug concentration in the aqueous humor over time (C_aqueous_) was then obtained through Equation (8) [38], considering the volume of the anterior chamber (V_aqueous_ = 0.250 cm^3^), the drug accumulation in the aqueous humor due to the release from the hydrogel (∂C/∂y) and the drug loss related to the aqueous humor renovation rate (Φ = 0.0025 mL/min) and through the cornea (P_cornea_A_cornea_), with A_cornea_ = 1.3 cm^2^. The boundary conditions state the symmetry at the center of the hydrogel (∂C/∂y = 0 at y = 0), the assumption of sink condition at the vitreous-IOL boundary (C = 0 at y = −h, i.e., on the posterior lens surface), and the equilibrium between the concentration on the anterior surface of the lens and the aqueous humor (C = KC_aqueous_ at y = h). The initial condition is the drug concentration in the lens at equilibrium (C = Ci ∀ y, at *t* = 0).

V_aqueous_ (∂C_aqueous_/∂t) = D A (∂C/∂y)_y = h_ − (P_cornea_A_cornea_ + Φ) C_aqueous_ (8)

### 2.9. Cytotoxicity

A2 and C2 hydrogel discs (*n* = 4) were loaded with both drugs as described above then rinsed, blotted and dried under vacuum for 24 h at 40 °C. Due to the thermosensitivity of corticosteroids [43], sterilization was then performed on dry discs by γ-radiation with a dose rate of 2.5 kGy/h at room temperature until a total dose of 25 kGy was reached [13]. Unloaded A2 and C2 hydrogel discs (*n* = 4) were also sterilized and tested.

Cytotoxicity was studied by indirect contact [28,44], placing the hydrogels in cell culture inserts (Transwell^®^, Corning, Glendale, AZ, USA; 12 mm diameter) and using human lens epithelial cells according to the ISO 10993-5: 2009 standard. Briefly, cells were cultured in a T75 flask with DMEM supplemented with 20% fetal bovine serum, 1% antibiotics (penicillin-streptomycin solution: 10,000 U/mL penicillin, 10 mg/mL streptomycin) and incubated at 37 °C in a humidified 5% CO_2_ incubator. Cell suspension, from passages 3 to 6, was seeded in each well of a 12-wells culture plate to obtain 1 × 10^5^ cells/well. Plates were incubated for 24 h at 37 °C under the same growth conditions. Meanwhile, drug-loaded and unloaded hydrogels were hydrated overnight in 1 mL of drug loading solution (sterile filtered 0.22 µm) or PBS, respectively. After proliferation, the culture medium was renovated, hydrogels were transferred into the inserts and these were placed in the wells. An additional 200 µL of fresh medium was added to ensure a complete immersion of the hydrogels. Negative (DMEM) and positive (DMEM with 10% DMSO) control wells (*n* = 4) were also prepared. After incubation for 24 h, inserts were removed, cells were rinsed with fresh PBS and incubated with MTT solution (MTT dissolved in serum-free DMEM at a concentration of 0.5 mg/mL, from a stock solution of 5 mg/mL MTT in PBS) for 3 further hours. Then, a formazan-dissolving solution (0.1% IGEPAL in isopropanol with hydrochloric acid 4 mM) was added to each well. Plates were shaken in a dark environment at room temperature for 1 h. The absorbance was measured at 595 nm in a microplate reader (Platos R 496, AMP Diagnostic, Graz, Austria). The relative quantification of cell viability was normalized to the negative control.

### 2.10. Statistical Analysis

Quantitative data are presented as the mean ± standard deviation. Statistical analysis was performed on Prism 8.0.1 software (GraphPad, San Diego, CA, USA) by *t*-test to compare two groups of samples and by one-way ANOVA to compare multiple groups with Tukey’s multiple comparisons post-hoc test. The normality of all variables was assessed by the Shapiro–Wilk test. The significance level was set at *p* < 0.05. The sample size was estimated to obtain average values with a maximum error of 7% and a confidence level of 95%. For mechanical tests, due to technical constraints, a maximum error of 14% with a confidence level of 90% was admitted.

## 3. Results and Discussion

### 3.1. Molecular Interaction Analysis

Hydrogels with a HEMA-BEM backbone were herein designed as IOL materials able to be simultaneously loaded with bromfenac sodium and dexamethasone sodium. APMA and AAm were selected as functional monomers after molecular docking analysis (Table 2), as they exhibited much stronger interaction parameters (i.e., higher free energy, E and lower dissociation constant, Ki) with the drug molecules if compared to the material backbone.

The free energy value describes the interaction between two molecules, namely the drug and the ligand (monomer). A more negative value of E indicates a higher interaction energy between the pair. The selected functional monomers APMA and AAm exhibit higher interaction with the drugs if compared to the backbone components of the hydrogels (HEMA and BEM). Therefore, the presence of APMA and AAm could result in the formation of suitable binding points for the drugs in the hydrogels. In particular, the interaction between the drugs and APMA presents more negative values, for both dexamethasone and bromfenac, and therefore higher affinity can be expected. Molecular docking analysis was previously used in various drug-release applications, with reported E values between −3.5 and −9.7 Kcal/mol for strong but still reversible drug-carrier interactions [45,46]. These values are comparable to the free energy, E, between APMA and the selected drugs.

The dissociation constant (Ki) provides information about the drug release from the material. It describes the interaction between the monomer (M) and the drug (D), by indicating the tendency towards the formation of two separate species (M + D) as opposed to the MD complex. A low Ki value indicates that the MD complex predominates over the species in separate (Equation (9)) [47]. Lower Ki values were obtained for the functional monomers APMA and AAm if compared to the backbone monomers.

Ki = [M][D]/[MD](9)

Less negative E values and high Ki values, and therefore low molecular affinity, were obtained by the interaction analysis of BEM with both drugs. The balance between the presence of BEM in the hydrogel backbone and the presence of the functional monomers may determine the drug release kinetics from the material.

### 3.2. Physical and Mechanical Characterization of the Unloaded Hydrogels

**Figure 2:**
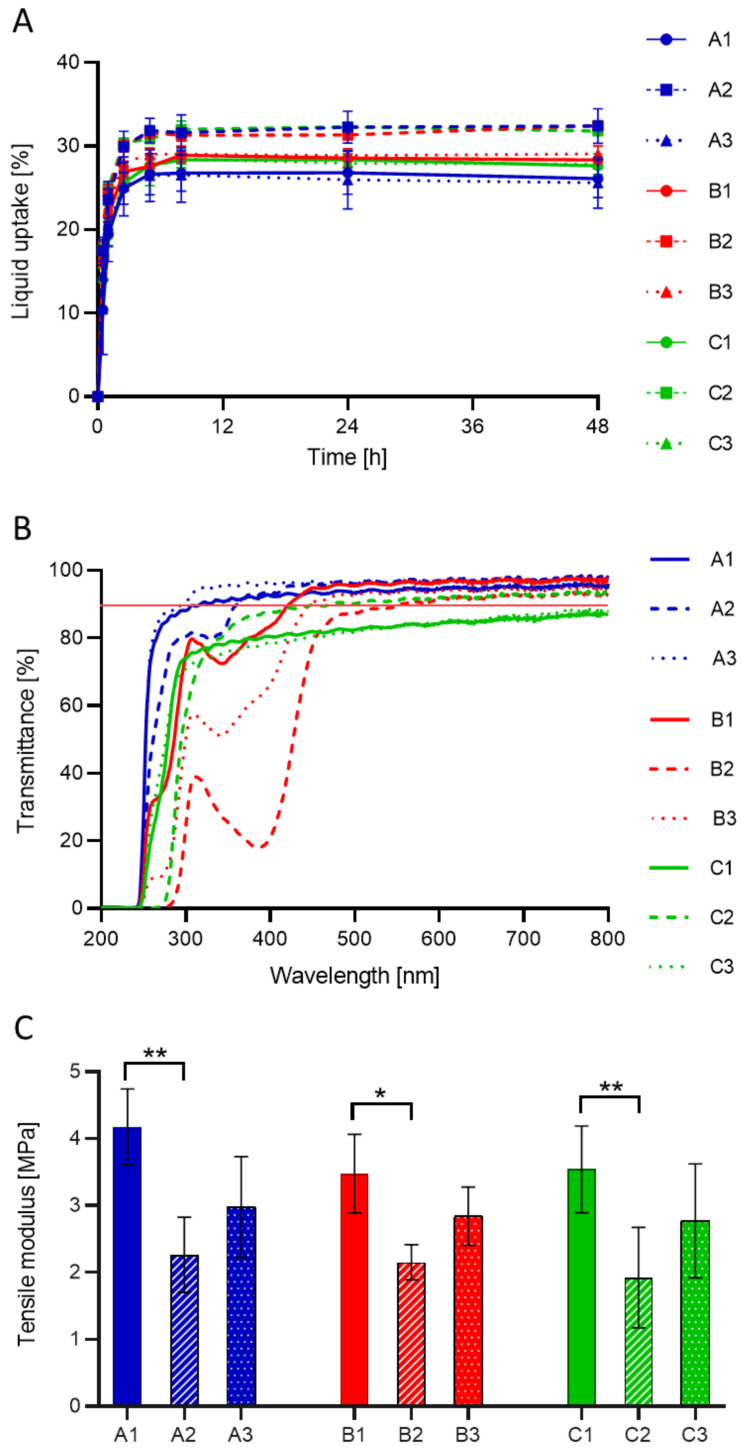
Physical and mechanical characterization of the designed hydrogels prior to drug-loading: liquid uptake (**A**), light transmittance (**B**) and mechanical properties (**C**). One-way ANOVA, * *p* < 0.05; ** *p* < 0.01. Hydrogel codes as in Table 1.

Prior to drug-loading, hydrogels were subjected to physical and mechanical characterization to prove their suitability as an IOL material. All hydrogel discs reached an equilibrium liquid content in the first 6 h of soaking (Figure 2A). Then, the liquid content remained stable for the subsequent time points. The highest liquid uptake was obtained for hydrogels containing APMA (i.e., A2, B2, C2 hydrogels, liquid uptake ≈ 32.2 %). The addition of AAm to the hydrogel composition (i.e., A3, B3, C3 hydrogels), on the contrary, did not cause any difference in the swelling behavior of the material. No statistical difference was evidenced between the drug-imprinted and non-imprinted hydrogels with the same monomer composition (*p* ≥ 0.2).

All hydrogels, except C1 and C3, exhibited a light transmittance higher than 90% at λ > 550 nm (Figure 2B), which was considered the minimum optical requirement for the materials [48]. As C1 and C3 hydrogels are both imprinted with dexamethasone sodium, the lower light transmission may have been caused by drug precipitation in the prepolymer mixture, forming drug clusters during polymerization, which were not dissolved during boiling. Differently, C2 hydrogel, also imprinted with dexamethasone, did not exhibit the same problem. The presence of APMA in C2 (Table 2) may have increased the solubility of dexamethasone sodium in the prepolymer mixture, thus avoiding precipitation. As can be noticed from the spectrum of A2 hydrogel, APMA also acted as a weak UV light filter for λ < 350 nm.

B1, B2 and B3 hydrogels, imprinted with bromfenac sodium, also exhibited light filter properties up to λ ≈ 450 nm. The presence of unremoved drug molecules in the polymers after the washing phase could justify the phenomenon, especially considering that the UV-block effect is more evident in the B2 hydrogel, functionalized with APMA, which has a stronger binding interaction with the drug (Table 2). In fact, the yellowish color of bromfenac sodium was observed in B2 hydrogels even after repeated washing.

The tensile moduli of the hydrated hydrogels are reported in Figure 2C. In general, all values (1.9–4.2 MPa) lie within the range reported for previously developed IOL materials and hydrophilic commercial lenses (i.e., 0.5–4 MPa [16,49,50]). Molecular imprinting did not significantly alter the mechanical properties of the hydrogels. The presence of APMA (in A2, B2, C2 hydrogels) lowered the tensile modulus of the material (≈2.1 MPa). This should also be related to the increased swelling of the hydrogels prepared with APMA (Figure 2A), which increases the intermolecular bonding between the hydrogel chains and water molecules, as opposed to hydrogel-hydrogel interactions.

The calculated values of the average mesh size are reported in the Supplementary Materials, Table S1. The average mesh size of A1 hydrogels was equal to 2.30 nm, which is coherent with previously reported values for HEMA-based hydrogels [51,52]. The mesh sizes of AAm-functionalized hydrogels were equal to 2.93, 2.76 and 2.87 nm for A3, B3 and C3 hydrogels, respectively. When functionalized with APMA, the mesh size increased up to 3.22, 3.31 and 3.47 nm for A2, B2 and C2 hydrogels, respectively. The larger mesh sizes of APMA-functionalized hydrogels are associated with higher liquid uptake and lower Young’s modulus (Figure 2A,C).

### 3.3. Drug Release In Vitro

**Figure 3:**
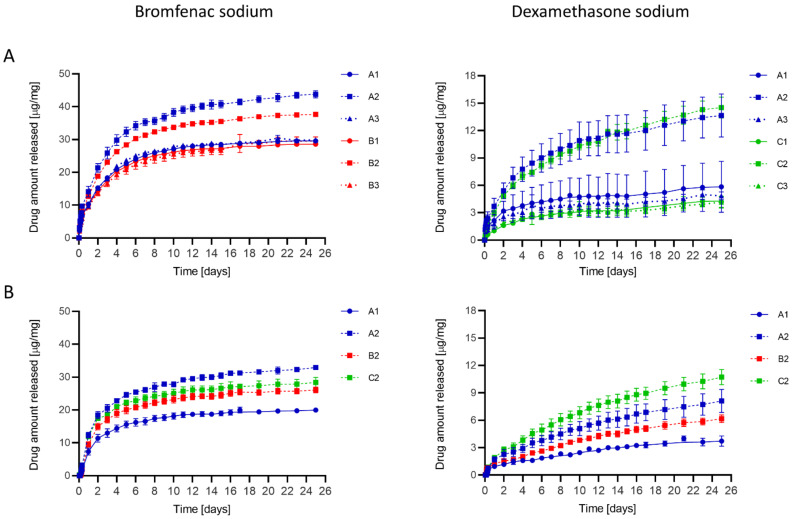
In vitro drug release from the hydrogel discs in 3 mL PBS, at 36 °C and 180 rpm after individual drug loading (**A**) and dual-drug loading (**B**) by soaking in drug solutions for 1 week at 36 °C.

Hydrogel discs were loaded with either bromfenac sodium (A1, A2, A3 and B1, B2, B3 hydrogels) or dexamethasone sodium (A1, A2, A3 and C1, C2, C3 hydrogels) and their release was subsequently tested in vitro for 4 weeks (Figure 3A). The use of APMA as a functional monomer (in A2, B2, C2 hydrogels) influenced the release profile: higher amounts of both drugs were released from functionalized hydrogels, and the release kinetics of dexamethasone were improved. Differently, functionalization with AAm (in A3, B3, C3 hydrogels) almost did not cause any difference in the release behavior if compared to non-functionalized hydrogels (A1, B1, C1 hydrogels). Molecular imprinting with bromfenac sodium on APMA-functionalized hydrogels (B2) decreased the drug amount released, while molecular imprinting with dexamethasone (C2) had practically no effect on the release of this drug.

APMA-functionalized hydrogels (A2, B2, C2) were selected as the most promising materials and were then subjected to dual-drug loading. An A1 hydrogel, not functionalized nor imprinted, was also tested as a control. Optimization of the loading conditions, namely soaking time (1, 2 or 4 weeks) and temperature (4 °C, 36 °C, 60 °C), was performed on the selected hydrogels. The influence of the loading parameters on light transmittance and drug release in vitro is reported in the Supplementary Materials Figures S1 and S2. Drug loading at 60 °C led to a decrease in transparency, probably due to thermal degradation of the drugs. This effect worsened with increased loading time. No alteration was reported in light transmittance when drug loading was performed at 4 °C or 36 °C. At 36 °C, the soaking time did not have a significant influence on the release profile, indicating that the hydrogel discs reached saturation within the first week of loading. Longer times were required to reach saturation for the bromfenac sodium when loading was performed at 4 °C. As A1 and A2 hydrogels loaded for 1 week at 60 °C satisfied the minimum optical requirements, their drug release was also studied. They led to the highest amount of dexamethasone released among the studied condition, but to the lowest of bromfenac. As a result, 1 week and 36 °C were selected as the optimal loading conditions. In future clinical applications, as soaking was performed at 36 °C for 1, 2 or 4 weeks without a significant influence on the transmittance and release profiles, the manufactured drug-loaded IOLs could be stored in their loading solution for at least 4 weeks prior to implantation.

The in vitro drug release profiles from A1, A2, B2 and C2 hydrogels loaded with both bromfenac and dexamethasone are reported in Figure 3B. The amount of bromfenac and dexamethasone released from the hydrogels decreased when loading was performed simultaneously, if compared to single-drug loading (Figure 3A), indicating a competitive behavior between the two drugs during the loading phase. In fact, both drugs are anionic in solution at pH 7.4 (Figure 1, pK_a_ 1.89 and 6.4 for dexamethasone sodium [53], pK_a_ 4.29 for bromfenac sodium [54]) and therefore target the same binding sites in the hydrogel network. As previously experienced with single-drug loading, A2 hydrogel released the highest amount of bromfenac. Imprinting with bromfenac (B2 hydrogel) resulted in a decreased amount of drug released compared to non-imprinted networks (A2 hydrogel) after both single and dual-drug loading (Figure 3). This is consistent with the presence of permanently-bonded bromfenac molecules in the material, as hypothesized in Section 3.2, which could occupy the binding sites of APMA thus, hindering further drug loading during the soaking phase. A different trend was observed for dexamethasone after dual-drug loading (Figure 3B), for which imprinting (C2 hydrogel) increased the drug amount released if compared to the addition of only APMA (A2 hydrogel). The E values modeled by molecular docking indicate that APMA forms weaker bonds with dexamethasone (E = −2.77 Kcal/mol) than with bromfenac (E = −3.45 Kcal/mol), which could facilitate drug removal during the washing step after polymerization, therefore leaving drug-shaped cavities in the hydrogels with oriented binding sites for the subsequent drug loading.

The diffusion of the drug molecules in the liquid phase of the hydrogels, and their affinity to the network, both contribute to the release mechanism. The presence of APMA as a functional monomer, or, in the case of imprinted networks, the orientation of its binding sites, increased the affinity between the drugs and the hydrogels, slowing down the release. Thus, the drug release mechanism from the hydrogels may be considered as an affinity-based delivery [55].

### 3.4. Drug Amount Loaded

The amounts of drugs loaded in hydrogels A1, A2, B2 and C2 after dual-drug loading are shown in Table 3. These results follow the same tendency of the previously described release profiles: the highest amounts of bromfenac and dexamethasone were loaded by hydrogels A2 and C2 (Table 3), respectively, which also released the highest amounts of these drugs (Figure 3B). The increased drug loading in APMA-functionalized hydrogels (A2, B2, C2), if compared to the A1 hydrogel (not functionalized nor imprinted), is consistent with the molecular docking analysis, which indicated a strong energy of interaction between APMA and the drugs (Table 2). APMA also influenced the physical properties of the hydrogel, leading to a higher water uptake (Figure 2A). Therefore, the increased amount of drug loaded into APMA-functionalized hydrogels could be the result of two concomitant effects: the higher drug affinity to the monomer and the higher swelling during the loading phase.

The amounts of loaded drugs were compared to the cumulative amounts of drugs released after 25 days of soaking in PBS, which corresponded to the last measured time points (Figure 3B). About 11–16% of the loaded bromfenac remained in the hydrogels after 25 days. As all release curves reached a plateau before the last time point, the unreleased drug could be trapped in the hydrogel and would therefore not be released in PBS. Another possibility is that the remaining drug would be slowly released at small concentrations, undetectable by the adopted spectroscopy method. The use of APMA as a functional monomer (in A2, B2, C2 hydrogels) did not have an effect on the percentage of unreleased bromfenac at the end of the release test.

Dexamethasone was entirely released from the A1 hydrogel after 25 days in PBS. As previously observed for bromfenac, the presence of APMA increased the amount of dexamethasone loaded. In the case of dexamethasone, however, the presence of the functional monomer influenced the release kinetics, and about 17–30% of the drug remained unreleased in A2, B2 and C2 hydrogels after 25 days in PBS. As the in vitro release curves from these APMA-functionalized hydrogels (Figure 3B) did not reach a plateau by the end of the test, the release of dexamethasone is expected to be sustained for more than 4 weeks in PBS.

### 3.5. Drug Permeability through the Cornea

The hydrogel discs that released the highest amount of bromfenac and dexamethasone (A2 and C2 hydrogels, respectively) were loaded with the two drugs simultaneously and placed in the donor chamber of Franz diffusion cells to evaluate the drug permeability through the cornea ex vivo.

As the amount of dexamethasone sodium permeated to the receptor chamber was below the HPLC quantification limit, it was not possible to calculate the permeability coefficient for the drug and only the results related to bromfenac sodium are shown (Supplementary Materials Figure S3, Table 4). The obtained permeability coefficient of bromfenac sodium (17–18 × 10^−7^ cm/s) was comparable to previously reported values (≈21 × 10^−7^ cm/s) for the free drug solution tested using the same experimental set-up [40]. Therefore, the permeability coefficients experimentally obtained with free drug solutions are expected to be valid also when hydrogel discs are used as drug carriers. Consequently, it is assumed that the permeability value reported in the literature for dexamethasone sodium in a free-drug solution (≈2.7 × 10^−7^ cm/s [56]) could also be valid for drug-eluting hydrogels, thus overcoming the experimental limitations found by the low drug amount released by the hydrogels during the permeability test.

### 3.6. Prediction of In Vivo Efficacy

A mathematical model was applied to predict the therapeutic efficacy of dual-drug loaded hydrogels after implantation as IOLs. The partition coefficient K and effective diffusivity D were calculated to be used as input parameters for the model, among others, and are reported in Table 5. The curve fittings for the determination of D values are reported in Supplementary Materials Figure S4.

Hydrogels prepared with APMA (A2, B2, C2 hydrogels) significantly decreased the D values of both drugs. Vacant binding sites of the functional monomer may interact with the drug molecules while they diffuse outside the polymer network, slowing down drug release. The increased affinity between the drugs and APMA-functionalized polymers is also evidenced in the calculated partition coefficient K (Table 5), which significantly increased in the presence of the functional monomer. Molecular imprinting, on the other hand, did not have any effect on the release kinetics.

**Figure 4:**
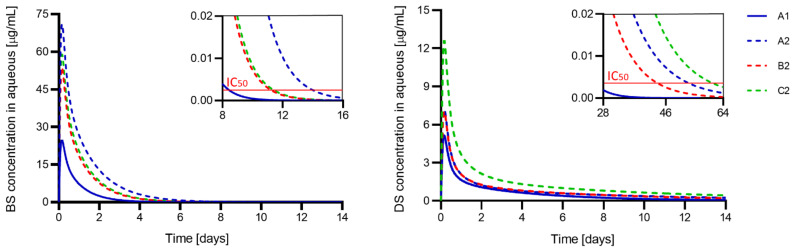
Estimation of the in vivo concentration of bromfenac sodium (BS, left) and dexamethasone sodium (DS, right) in the aqueous humor over time after the implantation of drug-loaded hydrogel discs as IOLs. The horizontal red line in the upper boxes indicates the IC_50_ concentration of each drug. Hydrogel codes as in Table 1.

The D values obtained for dexamethasone are one order of magnitude lower than those for bromfenac, which is reflected on both the release profiles in vitro (Figure 3) and the estimation of the drug concentration over time in vivo (Figure 4). The slower diffusion of dexamethasone, if compared to bromfenac, does not seem to be linked to a stronger affinity to the hydrogel monomers, as the partition coefficient K (Table 5), the free energy E (Table 2) and the total amount of drug loaded (Table 3) are inferior to the values obtained with bromfenac. The molecule of dexamethasone (Figure 1), however, presents a double negative charge in solution at pH 7.4 (pK_a_ 1.89 and 6.4 for the two potentially charged groups [53]), and could interact with two active sites in the hydrogel network. The fact that dexamethasone has a higher molecular weight (MW 516.42 g/mol) than bromfenac sodium (MW 356.15 g/mol) could also explain the slower drug diffusion during both the loading and the release processes, considering that the steric effects may be even more relevant in the case of the simultaneous diffusion of two drug molecules.

Bromfenac sodium is expected to be released by A2 hydrogels for the longest time (14 days) at therapeutic drug concentrations (i.e., above IC_50_ = 2.65 ng/mL [57]). The A2 hydrogel also exhibited the highest initial peak in drug concentration in the eye. The fastest exhaustion is expected in the A1 hydrogel, with a therapeutic efficacy of about 8 days only. No difference was observed between the B2 and C2 hydrogels in terms of K and D values, therapeutic efficacy over time and the initial peak of drug concentration.

The C2 hydrogel released dexamethasone sodium at therapeutic concentrations (i.e., above IC_50_ = 3.2 ng/mL [58]) for the longest time (8 weeks). The A2 and B2 hydrogels, which presented K and D values comparable to C2, are also expected to release dexamethasone for 6–7 weeks. The A1 hydrogel presented the shortest therapeutic efficacy (≈4 weeks), the lowest partition coefficient and the highest diffusivity value.

The current prophylaxis against PCME is usually performed with anti-inflammatory eye drops for 3–4 weeks after cataract surgery [6,8]. The therapy can be adjusted considering pre-existing conditions in the patients, such as the presence of risk factors for the development of PCME or sensibility to specific drugs. Different hydrogel compositions were herein proposed and could be selected depending on the required in vivo release profile (Figure 4). For example, as the use of steroids can be associated with IOP elevation [11], A2 hydrogel could be preferred over C2 hydrogel in patients affected by glaucoma: A2, in fact, maximizes the release of the non-steroidal drug bromfenac sodium, sustaining the release of dexamethasone at lower concentrations. C2 lenses, on the other hand, have the longest therapeutic effect and could be suitable in patients with other pre-existing risk factors for PCME, who need a prolonged prophylactic anti-inflammatory action (≈4–6 weeks after surgery [7]). In the current work, the influence of different concentrations of APMA was not investigated. However, it is possible that different degrees of functionalization with APMA could tune the drug affinity with the hydrogel and therefore the release profile.

### 3.7. Characterization of the Drug-Loaded Hydrogels

**Figure 5:**
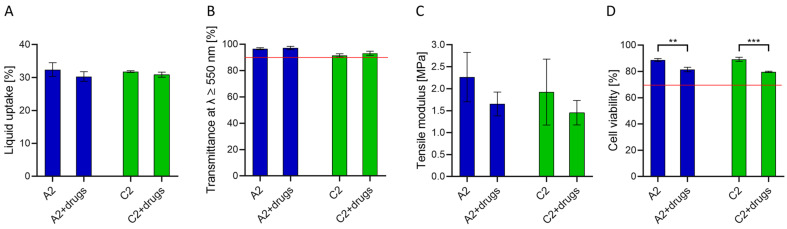
Characterization of the most adequate hydrogels after swelling in PBS (A2, C2) and after loading in a dual-drug solution (A2 + drugs, C2 + drugs): Liquid uptake (**A**), transmittance at λ ≥ 550 nm (**B**), tensile modulus (**C**) and cell viability (**D**). The minimum cell viability to assess non-toxicity was set at 70% (red line). *t*-test, ** *p* < 0.01, *** *p* < 0.001.

Based on the reported results, A2 and C2 hydrogels are expected to sustain the simultaneous release of bromfenac sodium and dexamethasone sodium at therapeutic concentrations in vivo for the longest time (Table 5, Figure 4) and therefore they were selected as the best materials for the prevention of PCME after cataract surgery. They were reevaluated regarding suitability as IOL materials in terms of physical, mechanical and biological characterization after dual-drug loading. The results were similar to those of unloaded hydrogels (Figure 5).

Hydrogel swelling in the dual-drug loading solution was comparable to the swelling obtained in PBS (Figure 5A), with a 31% average liquid uptake. Light transmittance in the longer visible range was not affected by the presence of the drugs, resulting in higher than 90% at λ ≥ 550 nm (Figure 5B), which is similar to the transmittance of commercially available IOLs [48]. The tensile modulus was not significantly different in both A2 (2.3 ± 0.6 vs. 1.6 ± 0.3 MPa) and C2 (1.9 ± 0.8 vs. 1.4 ± 0.3 MPa) hydrogels before and after loading, respectively (Figure 5C).

In vitro cell viability tests were performed using unloaded and dual-loaded discs to evaluate the biocompatibility of the material and the drug released. Cell viability was 88 ± 1% and 89 ± 2% for unloaded A2 and C2 hydrogels, and 81 ± 1% and 79 ± 1% for drug-loaded A2 and C2 hydrogels, respectively (Figure 5D), hence considered non-cytotoxic according to the ISO 10993-5: 2009 standard. Although a decrease in cell viability was observed in the presence of drugs, it should be considered that cell tests were performed without mimicking the continuous renovation of the aqueous humor, which is expected to reduce the time of contact of the drug and cytotoxicity in vivo. After eye implantation of drug-loaded hydrogels, a peak in drug concentration in the aqueous humor is expected in the first day (Figure 4), with maximum concentrations of dexamethasone and bromfenac equal to 12.6 µg/mL and 71.0 µg/mL from C2 and A2 hydrogels, respectively (Table 5). These values are much lower than the concentrations of currently used eye drops (900 µg/mL bromfenac collyrium, Yellox^®^ [59]; 1 mg/mL dexamethasone collyrium, Maxidex^®^ [60]) or intraocular injections (up to 1.1–1.6 mg/mL of dexamethasone [32,61]) and are, therefore, expected to be non-toxic for cells. As drug-loaded IOLs release the drug directly into the anterior chamber, drug loss associated with eye drops is avoided making lower drug concentrations sufficient to obtain a therapeutic effect. Indeed, less than 5% of the dose instilled with eye drops is expected to reach the aqueous humor [62,63,64]. The presence of an initial peak in drug concentration after implantation of the drug-loaded IOL could be beneficial for the intended treatment, as the administration of anti-inflammatory drugs is most required in the perioperative period [6,8].

Prior to being tested for cell viability in vitro, the hydrogels were sterilized in a dry state by γ-radiation. In the view of a future commercial application of the designed hydrogels, further tests are needed to evaluate the effect of sterilization on the material behavior over time, and the possibility of sterilizing the materials in the loading solution, for example using high hydrostatic pressure (HPP) [65].

Mathematical modeling was used in this study and constitutes a useful tool for the estimation of the efficacy of the devices in the early stage of product development. However, in vivo animal studies will have to precede any commercial application of the proposed hydrogels to evaluate the efficacy of the drug-eluting IOLs in post-surgical ocular conditions. Despite the significant achievements obtained in the last decade on the design of therapeutic IOLs, such devices are not currently present on the market and large-scale clinical trials are missing for a significant advance in the field [66].

## 4. Conclusions

Two different strategies, namely the addition of functional monomers and molecular imprinting, were herein investigated to tune the release profile of two anti-inflammatory drugs from HEMA-BEM hydrogels, with the aim of developing suitable materials for the fabrication of IOLs with prophylactic action against PCME. Bromfenac sodium and dexamethasone sodium were simultaneously loaded into the hydrogels. Drug-loaded hydrogels exhibited suitable physical and mechanical properties to be used as foldable intraocular lenses. The non-toxicity of the hydrogels and of the delivered drugs was assessed in vitro with human lens epithelial cells. The incorporation of APMA in the polymeric matrix improved the release kinetics of the drugs. Molecular imprinting with dexamethasone sodium increased the amount of dexamethasone released after dual-drug loading. Hydrogels A2 (non-imprinted containing APMA) and C2 (dexamethasone imprinted containing APMA) optimized the release of bromfenac and dexamethasone, respectively. Bromfenac is expected to be released at therapeutic concentrations in the aqueous humor for about 2 weeks after A2 hydrogel implantation, while C2 is expected to sustain the delivery of dexamethasone for up to 8 weeks. These durations are compatible with the current topical anti-inflammatory prophylaxis for PCME after cataract surgery. Dual-loaded IOLs made of the developed hydrogels will be able to efficiently substitute topical application, solve the compliance issues associated with the use of eye drops and address an unmet medical need for the prevention of PCME.

## Figures and Tables

**Table 1:** Composition of the nine formulations tested as potential IOL materials. A1, A2 and A3 are non-imprinted hydrogels. B1, B2, B3 are bromfenac-imprinted hydrogels. C1, C2 and C3 are dexamethasone-imprinted hydrogels.

Code	HEMA	BEM	APMA	AAm	Bromfenac Sodium	Dexamethasone Sodium	AIBN	EGDMA
A1	2.4 mL	600 µL					10 mM	80 mM
A2	2.4 mL	600 µL	100 mM				10 mM	80 mM
A3	2.4 mL	600 µL		100 mM			10 mM	80 mM
B1	2.4 mL	600 µL			25 mM		10 mM	80 mM
B2	2.4 mL	600 µL	100 mM		25 mM		10 mM	80 mM
B3	2.4 mL	600 µL		100 mM	25mM		10 mM	80 mM
C1	2.4 mL	600 µL				12.5 mM	10 mM	80 mM
C2	2.4 mL	600 µL	100 mM			12.5 mM	10 mM	80 mM
C3	2.4 mL	600 µL		100 mM		12.5 MM	10 mM	80 mM

**Table 2:** Free energy of interaction (E) and dissociation constant (Ki) of bromfenac sodium and dexamethasone sodium with HEMA, BEM, APMA and AAm monomers.

DRUG	HEMA		BEM		APMA		AAm	
	E [Kcal/mol]	Ki [mM]	E [Kcal/mol]	Ki [mM]	E [Kcal/mol]	Ki [mM]	E [Kcal/mol]	Ki [mM]
Dexamethasone sodium	−1.47	83.32	−1.10	156.55	−2.77	9.30	−2.06	30.74
Bromfenac sodium	−1.54	74.48	−1.27	117.06	−3.45	2.95	−2.20	24.43

**Table 3:** Amounts of bromfenac sodium and dexamethasone sodium (normalized per dry hydrogel weight) loaded by the hydrogels after dual-drug loading, amounts of drug released in vitro during 25 days, and percentage of unreleased drug after 25 days. Hydrogel codes as in Table 1.

Code	Bromfenac Sodium			Dexamethasone Sodium		
	Drug Amount Loaded (µg/mg)	Drug Released after 25 Days (µg/mg)	Unreleased Drug (%)	Drug Amount Loaded (µg/mg)	Drug Released after 25 Days (µg/mg)	Unreleased Drug (%)
A1	23 ± 1	20.0 ± 0.6	13	3.4 ± 0.1	3.7 ± 0.5	0
A2	37 ± 1	32.9 ± 0.6	11	11.2 ± 0.8	8 ± 1	28
B2	31 ± 2	26.1 ± 0.9	16	8.8 ± 0.7	6.2 ± 0.4	30
C2	32 ± 1	28 ± 2	11	13.0 ± 0.8	10.7 ± 0.8	17

**Table 4:** Cumulative mass of bromfenac sodium permeated, steady state flux J and permeability coefficient P_cornea_ obtained by ex-vivo test with drug-loaded A2 and C2 hydrogel discs. Hydrogel codes as in Table 1.

Code	Cumulative Mass Permeated (µg/cm^2^)	J (µg/cm^2^/h)	P_cornea_ × 10^7^ (cm/s)	R^2^
A2	2 ± 1	0.30 ± 0.04	18 ± 2	0.96 ± 0.03
C2	0.9 ± 0.4	0.27 ± 0.08	17 ± 5	0.93 ± 0.08

**Table 5:** Input parameters to the mathematical model (partition coefficient K and effective diffusivity D) for the prediction of the in vivo drug concentration in the aqueous humour, peak drug concentration and therapeutic lifespan of the de-signed hydrogels. Hydrogel codes as in Table 1.

	Bromfenac Sodium				Dexamethasone Sodium			
Code	K	D × 10^14^ [m^2^/s]	Peak Concentration [µg/mL]	Time with [Drug] > IC_50_	K	D × 10^14^ [m^2^/s]	Peak Concentration [µg/mL]	Time with [Drug] > IC_50_
A1	13.0 ± 0.8	26 ± 2	24.8	8 days	1.2 ± 0.2	5.7 ± 0.1	5.1	4 weeks
A2	51 ± 4	17.8 ± 0.6	71.0	14 days	2.6 ± 0.8	2.6 ± 0.6	7.2	7 weeks
B2	33.5 ± 0.6	21.6 ± 1.3	53.8	11 days	2 ± 1	3.2 ± 0.4	7.1	6 weeks
C2	37 ± 3	21.6 ± 1.3	59.0	11 days	5 ± 2	2.5 ± 0.6	12.6	8 weeks

## Data Availability

The authors confirm that the data supporting the findings of this study are available within the article and its Supplementary Materials. Raw data are available upon request.

## Supplementary Materials

The following are available online at https://www.mdpi.com/article/10.3390/pharmaceutics13070976/s1, Figure S1: Light transmittance, measured at λ = 550 nm, of A1, A2, B2 and C2 hydrogels after dual-drug loading with different soaking parameters; Figure S2: Cumulative amount of drug released from A1, A2, B2 and C2 hydrogels in vitro after dual-drug loading at different temperatures and soaking times; Figure S3: Linear regression of the cumulative mass of bromfenac sodium permeated through the cornea; Figure S4: Determination of the effective diffusivity (D) by curve-fitting of the cumulative amount of drug released from A1, A2, B2 and C2 hydrogels over time in vitro. Table S1: Parameters used for the calculation of the average mesh size ξ: density of the dry hydrogel (ρ), zero-frequency shear storage modulus (G′(0)) and volume fraction of the polymer in the swollen network (φ).
