# Supplementary material for: Drug-Loaded Hydrogels for Intraocular Lenses with Prophylactic Action against Pseudophakic Cystoid Macular Edema
Source: Pharmaceutics. 2021 Jun 28;13(7):976. doi: 10.3390/pharmaceutics13070976 (PMC8309109; doi:10.3390/pharmaceutics13070976)
Supplement: Supplementary file 1 [file pharmaceutics-13-00976-s001.zip › pharmaceutics-1271158-supplementary.pdf]

pharmaceutics

Supplementary Materials: Drug-Loaded Hydrogels for Intraocular Lenses with Prophylactic Action against Pseudophakic Cystoid Macular Edema

Nadia Toffoletto, Madalena Salema-Oom, Soledad Anguiano Igea, Carmen Alvarez-Lorenzo and Benilde Saramago, Ana Paula Serro

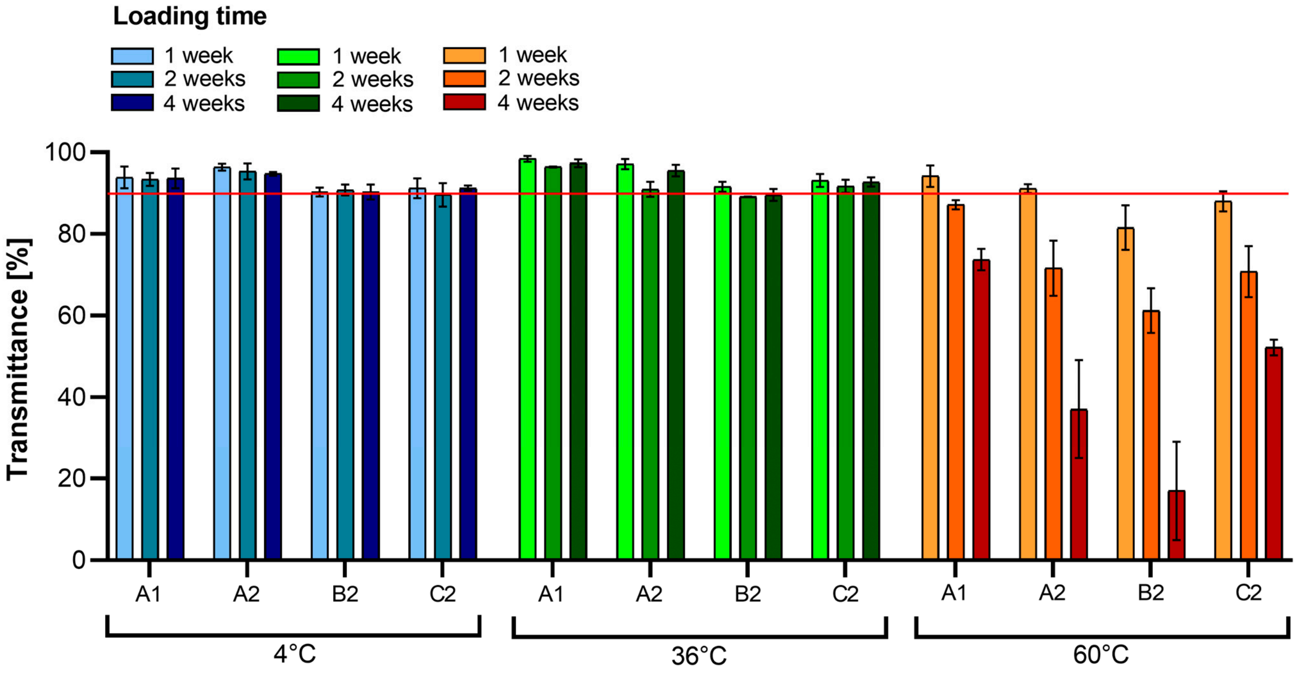

Figure S1. Light transmittance, measured at λ =550 nm, of A1, A2, B2 and C2 hydrogels after dual-drug loading with different soaking parameters: temperature (4 °C, 36 °C, 60 °C) and soaking time (1 week, 2 weeks, 3 weeks). The minimum optical requirement for the materials to be suitable as IOLs was set to 90% transmittance, indicated by the red line.

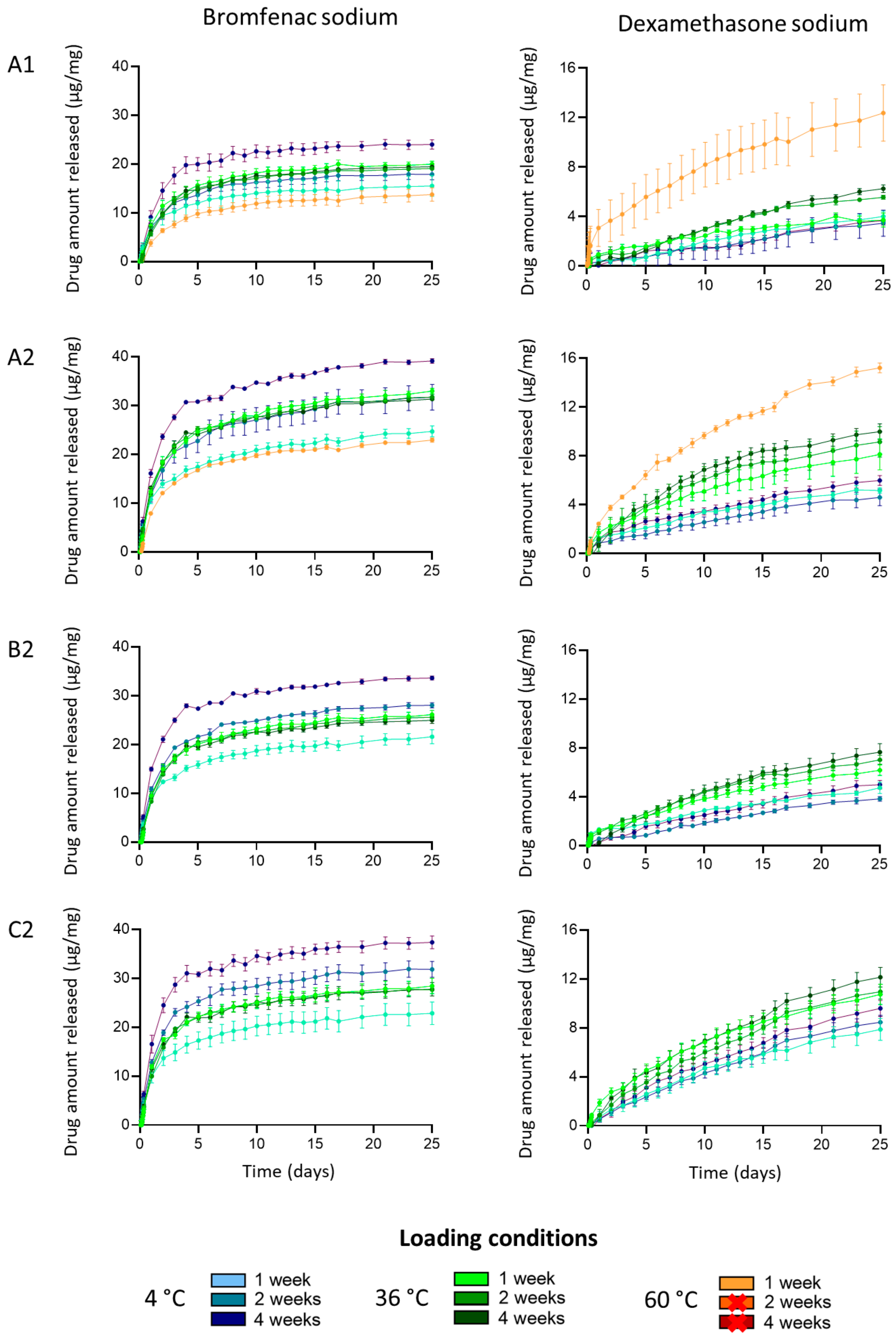

Figure S2. Cumulative amount of drug released from **A1**, **A2**, **B2** and **C2** hydrogels *in vitro* (normalized over sample weight) after dual-drug loading at different temperatures (4 °C, 36 °C, 60 °C) and soaking times (1 week, 2 weeks, 3 weeks). Among the samples loaded at 60 °C, only A1 and A2 loaded for 1 week satisfied the minimum optical requirements (Supplementary Figure S1) and were tested for drug release.

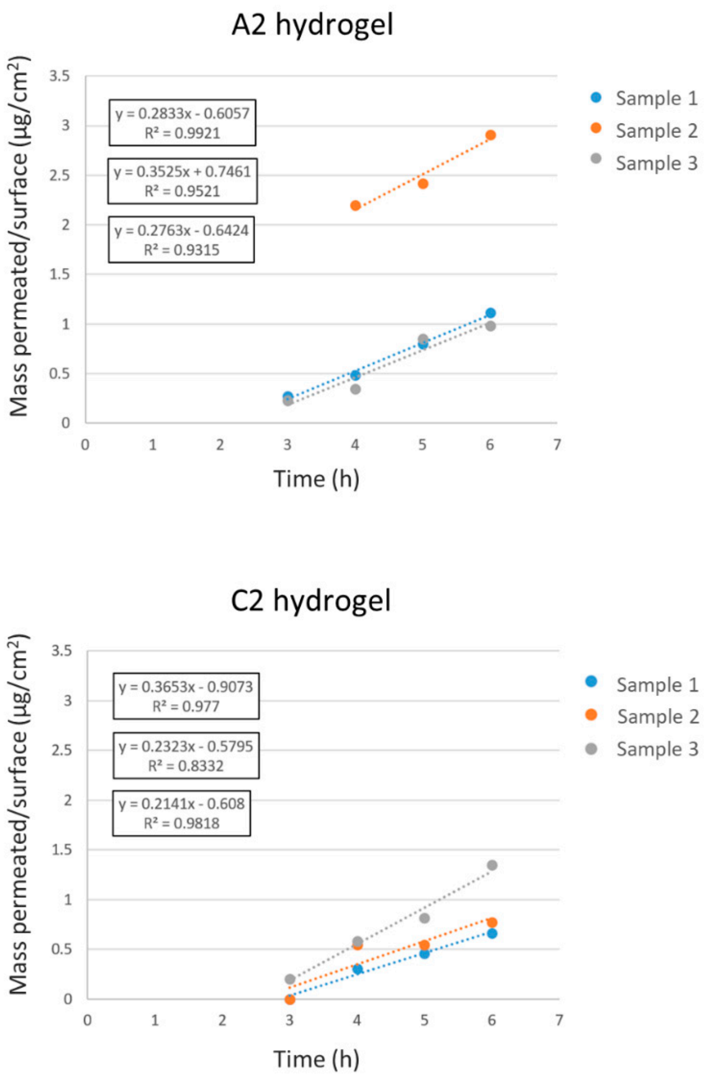

Figure S3. Linear regression of the cumulative mass of bromfenac sodium permeated through the cornea. The experiment was conducted placing dual-loaded A2 hydrogel (**top**) or C2 hydrogel (**bottom**) in the Franz cell's donor chamber. Excised porcine corneas were placed with the endothelium facing the donor chamber.

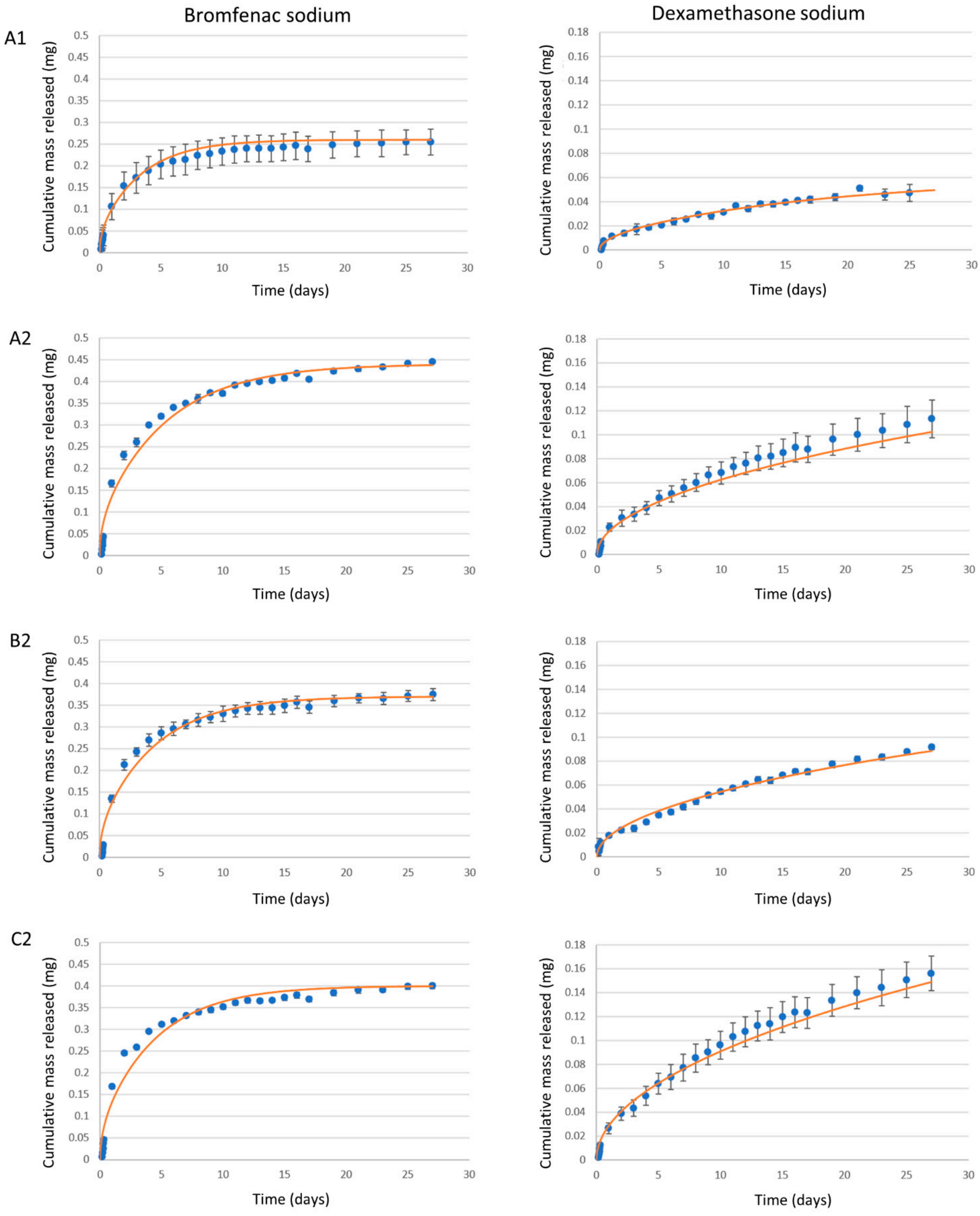

Figure S4. Determination of the effective diffusivity (D) by curve-fitting of the cumulative amount of drug released from A1, A2, B2 and C2 hydrogels over time *in vitro* to a one-dimensional diffusion equation.

Table S1. Parameters used for the calculation of the average mesh size ξ: density of the dry hydrogel (*ρ*), zero-frequency shear storage modulus (G′(0)) and volume fraction of the polymer in the swollen network (φ). The other values are: $l_{c\text{-}c}$ = 0.154 nm, C_F = 6.9 (assuming the value for polyHEMA [Peppas *et al.*, J Biomed Mater Res, 1985,19, 397-411]), and Mr = 134.62 g/mol for HEMA-BEM (80:20) hydrogels.

**Code**	***ρ* [g/cm³]**	**G′(0) [MPa]**	**φ [mm³/mm³]**	**ξ [nm]**
**A1**	1.197	1.392	0.87	2.30
**A2**	1.245	0.755	0.78	3.22
**A3**	1.339	0.992	0.70	2.93
**B1**	1.157	1.159	0.85	2.48
**B2**	1.261	0.717	0.80	3.31
**B3**	1.171	0.948	0.88	2.76
**C1**	1.268	1.181	0.80	2.59
**C2**	1.239	0.641	0.80	3.47
**C3**	1.231	0.924	0.84	2.87
